# Herbo-mineral formulation, Divya-Swasari-Vati averts SARS-CoV-2 pseudovirus entry into human alveolar epithelial cells by interfering with spike protein-ACE 2 interaction and IL-6/TNF-α /NF-κB signaling

**DOI:** 10.3389/fphar.2022.1024830

**Published:** 2022-10-26

**Authors:** Acharya Balkrishna, Sudeep Goswami, Hoshiyar Singh, Vivek Gohel, Rishabh Dev, Swati Haldar, Anurag Varshney

**Affiliations:** ^1^ Drug Discovery and Development Division, Patanjali Research Institute, Haridwar, Uttarakhand, India; ^2^ Department of Allied and Applied Sciences, University of Patanjali, Haridwar, Uttarakhand, India; ^3^ Special Centre for Systems Medicine, Jawaharlal Nehru University, New Delhi, India

**Keywords:** Divya-Swasari-Vati (DSV), SARS-CoV-2, pseudovirus, spike protein, ACE 2, proinflammatory cytokines

## Abstract

The herbo-mineral formulation, Divya-Swasari-Vati (DSV), is a well-known Ayurvedic medication for respiratory ailments. In a recent pre-clinical study, DSV rescued humanized zebrafish from SARS-CoV-2 S-protein-induced pathologies. This merited for an independent evaluation of DSV as a SARS-CoV-2 entry inhibitor in the human host cell and its effectiveness in ameliorating associated cytokine production. The ELISA-based protein-protein interaction study showed that DSV inhibited the interactions of recombinant human ACE 2 with three different variants of S proteins, namely, S^mut 1^ (the first reported variant), S^mut 2^ (W436R variant) and S^mut 3^ (D614G variant). Entry of recombinant vesicular stomatitis SARS-CoV-2 (VSVppSARS-2S) pseudovirus, having firefly luciferase and EGFP reporters, was assessed through luciferase assay and fluorescent microscopy. DSV exhibited dose-dependent inhibition of VSVppSARS-2S pseudovirus entry into human lung epithelial A549 cells and also suppressed elevated levels of secreted pro-inflammatory cytokines such as interleukin-6 (IL-6), interleukin-1β (IL-1β) and tumor necrosis factor-α (TNF-α) induced by viral infection mimicking Poly I:C-, S-protein- and VSVppSARS-2S pseudovirus. In human immune cells, DSV also moderated TNF-α-mediated NF-κB induction, in a dose-dependent manner. The observed anti-viral effect of DSV against SARS-CoV-2 is attributable to the presence of different metabolites Summarily, the observations from this study biochemically demonstrated that DSV interfered with the interaction between SARS-CoV-2 S-protein and human ACE 2 receptor which consequently, inhibited viral entry into the host cells and concomitant induction of inflammatory response.

## Introduction

The SARS-CoV-2 virus-led COVID-19 pandemic has dramatically impacted global demographics, causing ∼0.56 billion confirmed cases and ∼6.3 million deaths as of 15 July 2022 (World Health Organization, 2022). It emerged as the most menacing global healthcare catastrophe since the 1918 influenza pandemic. Acute COVID-19 cases involved severe clinical manifestations that involve hospitalization and mortality (Nalbandian et al., 2021). A gamut of factors such as highly contagious nature, rapid genetic evolution through mutations, and limited early testing facility contributed to the far greater epidemiological spread of the SARS-CoV-2 compared to its predecessors, SARS-CoV and MERS-CoV back in 2002 and 2012 respectively (Hu et al., 2021). The lack of treatment makes the management of COVID-19 very challenging (Gandhi et al., 2020). Mild to moderate COVID-19 symptoms, such as fever, cough, dyspnoea and fatigue, overlap with several non-lethal ailments (Fara et al., 2020). Thus, the management of asymptomatic and mild to moderate COVID-19 primarily relied on molecular diagnostics such as RT-PCR for viral identification. This formidable situation, barely managed by social distancing and enforcement of personal and community hygiene, underlines the unmet need for developing effective anti-viral therapeutics.

SARS-CoV-2 is an enveloped, positive-sense single-stranded RNA virus that belongs to the *Coronaviridae* family. Like the influenza virus, it is also zoonotic and invades alveolar epithelial and endothelial cells and inflicts severe respiratory illness on affected individuals (Hoffmann et al., 2020b). To understand the molecular mechanism behind this infection, the interaction between SARS-CoV-2 spike surface glycoprotein (S protein) and host ACE 2 receptor studied through atomic force microscopy (AFM) has been reported previously (Yang J. et al., 2020). The host-viral interaction was also explored through molecular dynamic simulation (Ali and Vijayan, 2020). The ability of the SARS-CoV-2 S protein to detect and interact with ACE 2 receptors of the target host cell and the functions of proteases like TMPRSS2 for the viral invasion have been reported in several other studies (Hoffmann et al., 2020b; Hu et al., 2021; Jackson et al., 2022). The predisposition of this virus to cause upper and lower respiratory tract infections are well known now. An abundance of ACE 2 receptors in pneumocytes (type I and type II epithelial cells), alveolar macrophages, and enterocytes make these cells favorable primary targets for SARS-CoV-2 infection. The virus enters and replicates, and mature virions are then released to infect new target cells. Besides human lung cells, SARS-CoV-2 can infect other non-human mammalian cells such as mucosal cells of intestines, tubular epithelial cells of kidneys, epithelial cells of renal tubules, cerebral neurons, and immune cells, where ACE 2 receptors are expressed (Fara et al., 2020). There are two subunits of S protein (S1 and S2) with an arginine-rich furin cleavage site in between. The host-cell mediated proteasomal cleavage of this site is indispensable for viral entry into human lung cells and subsequent manifestation of infection (Hoffmann et al., 2020a). The S1 subunit at the N-terminal of the S protein harbours the Receptor Binding Domain (RBD) that enables the S protein to interact with the host cell ACE 2 receptor, whereas the S2 subunit mediates viral-host cell membrane fusion. This receptor-mediated endocytic route of viral entry into the host cell also requires priming of the S protein by host serine protease TMPRSS2, cathepsin L, and furin (Du et al., 2009). However, targeting host proteases involved in the viral entry is likely to cause undesirable side effects. Therefore, specifically limiting S protein-ACE 2 interaction would be a more appropriate method to hinder viral entry into the cell.

The SARS-CoV-2 infection triggers a dramatic host inflammatory response. It involves an overproduction of soluble pro-inflammatory cytokines, such as IL-1β, IL-6, IL-12, type I and II interferons, IFN-γ, TNF-α, monocyte chemoattractant protein-1 (MCP-1), and macrophage inflammatory protein-1A (MIP-1A). A cascade of biochemical events sparks downstream activation of (JAK)-STAT3, JAK-SHP-2-MAP kinase and NF-κB-dependent signalling pathways. This uncontrolled hyper-inflammatory response accelerates toward cytokine storm syndrome (CSS), an apparent hallmark of the COVID-19 malady. Although intended for viral clearance, this overdriven host immune response results in self-annihilation leading to an acute respiratory distress syndrome (ARDS) in the case of severe COVID-19 patients. The severity of COVID-19 prognosis begins with classical signs of inflammation followed by apoptosis of epithelial cells, reduced peripheral oxygen saturation, hemorrhage, vascular leakages, tissue damage, elevated C-reactive proteins and multiple-organ malfunctioning. The aforementioned unregulated clinical manifestations mimic the state of transient autoimmune disorder (Fara et al., 2020; Tang et al., 2020). To combat SARS-CoV-2 induced hypercytokinemia and CSS associated with severe COVID-19, inhibitors of pro-inflammatory STAT1 and NF-κB signaling pathways, several anti-inflammatory drugs, and immunomodulatory agents, like, IL-1β, IL-6 antagonists and TNF-α blockers, were widely used to control the infection (Mehta et al., 2020; Ye et al., 2020; Rabaan et al., 2021). However, indiscriminate use of these agents weakened the host immune system to such an extent that COVID-19-associated mucormycosis (CAM), a fungal endemic was reported in India in the wake of the second wave of the viral pandemic (Patel et al., 2021).

Based on the understanding gained on COVID-19 etiology through previous investigations, it is postulated that a potential anti-COVID therapeutic strategy should aim for developing a drug with multi-targeted functionality. The potential therapeutic approaches are anticipated to disrupt the viral pathway/s as well as block SARS-CoV-2 from fusing with or entering the host cells, hence reducing the overall invading viral load and associated virus-induced CSS in the host cells. In case some infective virions get inside the host cell, the drug should ameliorate viral infection-induced acute pathophysiological conditions by downregulating undesirable boost in cytokine production. Such delayed immune response will rescue host cells from the detrimental autoimmune impact of the CSS caused by a viral infection which inherently minimizes the risk of unhindered proliferation and spread of the infection and alleviates patients from hospitalization (Felsenstein et al., 2020). Several publications, based on computational studies, speculated on the promising potential of metabolites against SARS-CoV-2, resulting in a shift in attention to different traditional systems of medicines. Ethnomedicinal herbs containing natural bioactive molecules of renowned broad spectrum medicinal significance with no side effects could be explored to fill this void in SARS-CoV-2 therapeutic stratagems (Abubakar et al., 2021).

Ayurveda, the system of Traditional Indian Medicine (TIM), has recommended formulations for different types of ailments. Divya-Swasari-Vati (DSV), consisting of various parts of nine herbs and seven classical mineral preparations, called “*bhasma*” (Table 1), is an Ayurvedic herbo-mineral formulation prescribed for respiratory ailments, such as cold, cough, bronchitis, and asthma. In order to ensure reproducibility of the subsequent pharmacological studies on DSV as required by the “Consensus statement on the Phytochemical Characterisation of Medicinal Plant extracts” (ConPhyMP) (Heinrich et al., 2022), a thorough process optimization has been done for consistent detection of marker metabolites in DSV across different batches (Balkrishna et al., 2021c; Balkrishna et al., 2021d). DSV is rich in several metabolites, such as eugenol, piperine, glycyrrhizin, gallic acid, 6-gingerol, methyl gallate, ellagic acid, glabridin, cinnamic acid, protocatechuic acid and coumarin (Table 2) (Balkrishna et al., 2020c; Balkrishna et al., 2021c). The pharmacological significance of the metabolites present in DSV, particularly their anti-inflammatory properties, are well documented (Semaming et al., 2015; Semwal et al., 2015; Correa et al., 2016; Graebin, 2018; Rios et al., 2018; Kahkeshani et al., 2019; Ruwizhi and Aderibigbe, 2020; Li C.-X. et al., 2021; Nisar et al., 2021; Sharifi-Rad et al., 2021; Tripathi et al., 2022). Recently, DSV has also been reported to ameliorate S protein-induced inflammatory response in A549 xenotransplanted zebrafish by reducing IL-6 and TNF-α, pro-inflammatory cytokine levels and rescuing fever-driven swimming behavioural patterns (Balkrishna et al., 2020c). Converging evidence warrant mechanistic investigation of the anti-viral and immunomodulatory activities of DSV against SARS-CoV-2 pathologies under *in vitro* experimental setup.

**TABLE 1:** Different components (botanical drugs and minerals) used in Divya-Swasari-Vati (DSV) tablet formulation^a^.

Sl. No.	Herbal (botanical drugs) components					% In each DSV tablet^b^
	Scientific name	English name	Sanskrit binomial name	Hindi name	Plant part used	
1.	*Glycyrrhiza glabra* L.	Licorice	Yas.ṭimadhukaḥ aroma (यष्टिमधुक: अरोमा)	Mulethi	Root	11.85
2.	*Pistacia chinensis* subsp*. integerrima* (J. L. Stewart) Rech. f.	Zebrawood	Karkaṭaśṛṅgakam raktapallavam (कर्कटशृङ्गकम् रक्तपल्लवम्)	Kakadasingi	Gall	11.66
3.	*Cressa cretica* L.	Cretan alkaliweed	Rudantakaḥ caṇapatraḥ (रुदन्तक: चणपत्र)	Rudanti	Fruit	11.66
4.	*Piper nigrum* L.	Black pepper	Kaṇikā maricā (कणिका मरिचा)	Marich	Fruit	7.77
5.	*Piper longum* L.	Long pepper	Kaṇikā pippalī (कणिका पिप्पली)	Choti pippal	Fruit	7.77
6.	*Zingiber officinale* Roscoe	Ginger	Ārdrakam sitaus.ṭham (आर्द्रकम् सितौष्ठम्)	Sounth	Rhizome	7.77
7.	*Cinnamomum verum* J. Presl	Cinnamon	Gandhajātakam tvak (गन्धजातकम् त्वक्)	Dalchini	Bark	5.92
8.	*Syzygium aromaticum* (L.) Merr. & L. M. Perry	Clove	Jambukaḥ lavaṅgaḥ (जम्बुक: लवङ्ग)	Lavang	Flower bud	5.92
9.	*Anacyclus pyrethrum* (L.) Lag.	Spanish chamomile	Ākārakarabhakaḥ chinnapatraḥ (आकारकरभक: छिन्नपत्र)	Akarkara	Root	5.92

Mineral components						
	Sanskrit names	Description		
10.	Mukta- Shukti Bhasma	Herbally processed ash from calcined shell of pearl oyster (*Pinctada fucata*)				2.33
11.	Godanti Bhasma	Herbally processed ash from rich gypsum				2.33
12.	Kapardak Bhasma	Herbally processed ash from calcined cowry shell of *Cypraea moneta*				2.33
13.	Abhrak Bhasma	Herbally processed ash from calcined mica				2.33
14.	Sphatika Bhasma	Herbally processed ash from calcined form of alum				2.33
15.	Praval Pishti	Coral calcium powder processed with rose water				2.33
16.	Tankan Bhasma	Herbally processed ash from calcined borax				2.33

^a^ Adapted from Table 1 of (Balkrishna et al., 2021c; Balkrishna et al., 2021d).

^b^ Each DSV tablet weighs 540 mg.

**TABLE 2:** Metabolites present in Divya-Swasari-Vati (DSV) tablet formulation^a^.

Sl. No.	Name of the metabolite	Amount (μg/tablet^b^)	Molecular structure
1.	Eugenol	3149.28	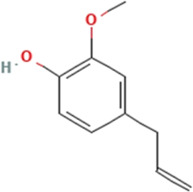
2.	Piperine	3080.70	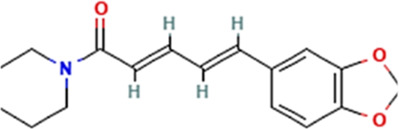
3.	Glycyrrhizin	1949.94	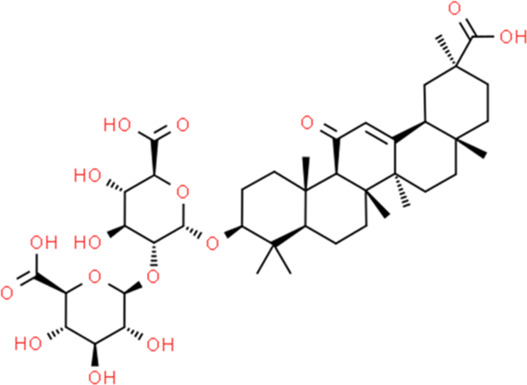
4.	Gallic acid	1837.08	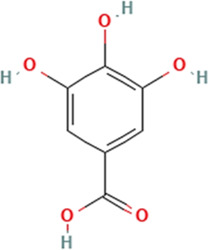
5.	6-Gingerol	255.96	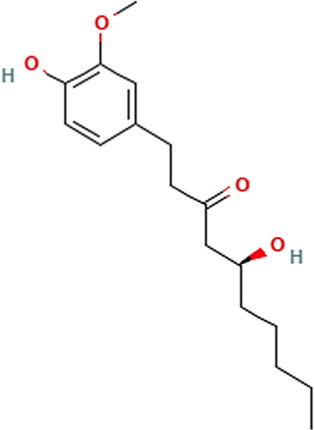
6.	Methyl gallate	150.12	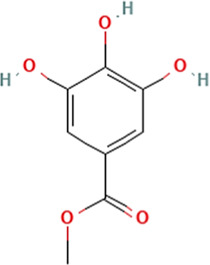
7.	Ellagic acid	150.12	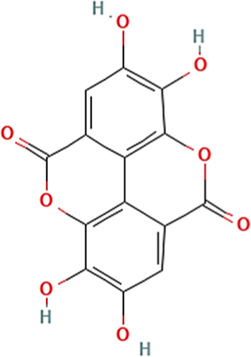
8.	Glabridin	116.10	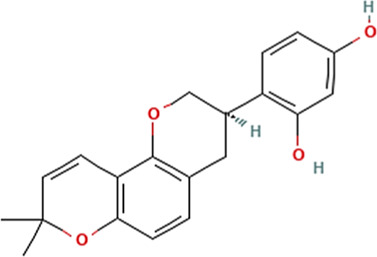
9.	Cinnamic acid	16.20	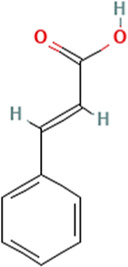
10.	Protocatechuic acid	14.04	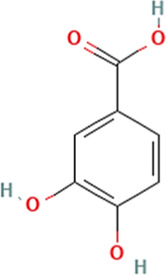
11.	Coumarin	7.02	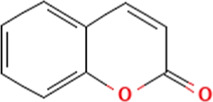

^a^ Adapted from Table 7 of (Balkrishna et al., 2020c). Chemical structures have been sourced from www.pubchem.com and/or www.chemspider.com.

^b^ Each DSV tablet weighs 540 mg.

Therefore, in the present study, DSV was evaluated for its inhibitory potency against SARS-CoV-2 pseudovirus entry into human lung cells and its concomitant immune response. The present investigation was designed to evaluate the prospects of DSV in curbing human ACE 2 and S-protein interaction through an ELISA-based biochemical assay. Furthermore, the efficacy of DSV inhibiting the entry of SARS-CoV-2 S protein pseudotyped virus into ACE 2 expressing human alveolar epithelial A549 cells was also investigated. Furthermore, the molecular pathway targeted by DSV to ameliorate COVID-19 pathologies was explored using reporter cell lines. The effectivity of DSV as an anti-viral, in general, was investigated in Poly I:C-induced THP-1 (human leukemia monocytic cells). The immunomodulatory effect of DSV was confirmed on SARS-CoV-2 S protein- and pseudovirus-induced cytokine response in human alveolar epithelial cells.

## Materials and methods

### Chemicals, reagents, cell lines and cell culture

DSV (Batch No. #A-SWV047, expiry May 2023) was obtained from Divya Pharmacy (Haridwar, Uttarakhand, India). Dulbecco’s Modified Eagle’s Medium (DMEM) (Catalog #12100046), Roswell Park Memorial Institute Medium 1640 (RPMI 1640) (Catalog #31800022) and Lipofectamine 3000 transfection kit (Catalog #L3000008) were procured from Thermo Fisher Scientific (Waltham, MA, United States). Fetal Bovine Serum (FBS) (Catalog #RM9955), Trypsin Phosphate Versene Glucose (TPVG) solution and Alamar Blue reagent were purchased from HiMedia Laboratories Pvt. Ltd. (Mumbai, Maharashtra, India). Penicillin-Streptomycin mixture (Catalog #P4333) and Poly I:C (Catalog #P1530) were obtained from Sigma-Aldrich (St. Louis, MO, United States). ELISA kits for interleukin-6 (IL-6) (Catalog #555220), interleukin-1 beta (IL-1β) (Catalog #557953), and tumor necrosis factor-alpha (TNF α) (Catalog #555212) were procured from BD Biosciences (San Jose, CA, United States). Purified SARS-CoV-2 Spike (S) proteins and purified human ACE 2 protein were obtained from Sino Biological Inc. (Beijing, China). ELISA-based SARS-CoV-2 inhibitor screening kit (Catalog #EP-105) was purchased from AcroBiosytems (Newark, DE, United States). Streptavidin conjugated horse-radish peroxidase (streptavidin-HRP) along with 3.3′,5,5′-Tetramethylbenzidine (TMB) (Catalog number #555214) were obtained from BD bioscience (San Diego, CA, United States). Luciferase assay substrate (Catalog #E151A), Luciferase assay buffer (Catalog #E152A) and 5X cell culture lysis reagent (Catalog #E153A) were purchased from Promega Corp. (Madison, WI, United States). All other chemicals and reagents were from Sigma-Aldrich (St. Louis, MO, United States) unless otherwise stated. Human alveolar epithelial (A549), human leukemia monocytic cell line (THP-1) and human kidney epithelial (HEK293) cell lines were procured from the American Type Culture Collection (ATCC) licensed cell repository at the National Centre for Cell Science (NCCS, Pune, India), whereas THP1-Blue NF-κB reporter cell line was obtained from InvivoGen (San Diego, CA, United States). A549 and HEK293 cells were cultured in DMEM supplemented with 10% (v/v) FBS, penicillin-streptomycin mixture (100 μg/ml) at 37°C under a humidified atmosphere containing 5% CO_2_ in a cell culture incubator. NF-κB reporter cells were cultured in RPMI 1640 medium under similar growth conditions as described above.

### Cell viability assay

Cytosafety of DSV was analysed through *in vitro* cell viability assay through Alamar Blue staining. In brief, 3 × 10^4^ PMA differentiated THP-1 cells were seeded in 96 well plate in triplicate and grown overnight. Cells were further incubated for 24 h in the presence of different concentrations of DSV (1, 3, 10, 30, 100 and 300 μg/ml) in a total volume of 100 μl in each well. DSV-untreated control samples were also included. Alamar Blue (10 μl) at a final concentration of 0.15 mg/ml was added to each well and incubated for a period of ∼3 h for the characteristic pink colour to develop in the control samples as per the manufacturer’s protocol. Fluorescence was measured at an emission of 590 nm and excitation of 560 nm, using an Envision microplate reader (PerkinElmer, Waltham, MA, United States), followed by the calculation of percent (%) cell viability considering 100% viability in the control sample.

### Quantification of poly I:C-induced cytokine activation in human leukemia monocytic cell line

For this assay, Poly I:C stock was prepared in molecular grade water and heated at 70°C for 10 min in PCR blocks and gradually allowed to cool. Meanwhile, PMA differentiated THP-1 cells were seeded at a cell density of 3 × 10^4^ cells/well. Poly I:C (10 μg/ml) was added to THP-1 cells, which were pre-incubated for 16 h, with varying concentrations of DSV (1, 3, 10, 30 μg/ml). Poly I:C-uninduced and induced THP-1 cells without DSV treatment were considered normal and disease controls, respectively. After 24 h co-treatment with Poly I:C and DSV, levels of secreted pro-inflammatory cytokines, namely IL-6 and TNF-α were estimated in the supernatants of the cultured THP-1 cells by using specific ELISA kits for each of these cytokines, as per the manufacturer’s protocol. Absorbance was measured at 450 nm using the Envision microplate reader (Perkin Elmer, United States).

### Enzyme-linked immunoassay-based biochemical assessment to detect the interaction between SARS-CoV-2 spike protein and human angiotensin converting enzyme 2

Three different variants of SARS-CoV-2 spike (S) proteins namely, S^mut1^ (Catalog #40592-V08B), S^mut2^ (Catalog #40592-V08H9), and S^mut3^ (Catalog #40591-V08H3) were analyzed quantitatively for their interactions with recombinant human ACE 2 protein (hACE 2) (Catalog #10108- H08H-B), through an ELISA-based SARS-CoV-2 inhibitor screening kit following the manufacturer’s protocol as reported earlier (Balkrishna et al., 2021a). Briefly, each well of the ELISA plate was incubated separately with 25 ng of different variants of S proteins for coating. Thereafter, following thorough washings, 62.5 ng of purified biotinylated human ACE 2 protein was added to each well in a total volume of 100 μl in the presence of different concentrations of DSV (0.001, 0.003, 0.01, 0.03, 0.1, 0.3, 1.0, 3.0, 10, and 30 μg/ml). Subsequently, samples were incubated for 30 min. Negative control included only ACE 2 and S proteins without any exposure to DSV. Positive control was supplied along with the kit and included a chemical inhibitor (Catalog # AC2-NA005; AcroBiosytems, Newark, DE, United States) against ACE 2-S protein interaction. With the subsequent addition of streptavidin-HRP along with the substrate, TMB helped in quantifying the amount of ACE 2 interacting with S proteins. The absorbance of the oxidized TMB was measured at 450 nm using an Envision microplate reader (Perkin Elmer, United States). ACE 2 and S protein interactions were calculated as percentages (%) with respect to the untreated/negative control group showing 100% interaction.

### Estimation of the cytokine response induced by different S proteins in alveolar epithelial A549 cells

Cytokine responses elicited by different variants of S protein in A549 cells were determined as mentioned earlier (Balkrishna et al., 2021a). A549 cells were grown to 50–60% confluent. The cells were then exposed to 8,000 ng/ml of S^mut1^ and S^mut2^ and 20,000 ng/ml of S^mut3^ for 48 h, after which the levels of the pro-inflammatory cytokines, IL-6, TNF-α, and IL-1β secreted in the supernatants of cultured cells were measured by using specific ELISA kits for each of these cytokines, following manufacturer’s protocol. The Envision microplate reader (Perkin Elmer, United States) measured absorbance at 450 nm.

### Vesicular stomatitis virus pseudotyping with SARS-CoV-2 S protein

SARS-CoV-2 S protein pseudotyping of vesicular stomatitis virus (VSV) was conducted according to previous reports (Whitt, 2010; Hoffmann et al., 2020b). Briefly, 24 h before infection with replication-deficient VSV*ΔG-FLuc viruses, HEK293 cells were co-transfected with helper plasmids encoding VSV N, P, and L proteins of VSV and SARS-CoV-2S protein expressing pCG1-SARS-2S plasmid. VSV*ΔG-FLuc viruses, set of helper and pCG1-SARS-2S plasmids were kind gifts from Prof. Stefan Pöhlmann (Deutsches Primatenzentrum GmbH, Leibniz-Institut für Primatenforschung, Kellnerweg 4, 37,077 Göttingen, Germany), and received with approval from the Institutional Biosafety Committee of Patanjali Research Institute (vide approval # IBSC/PRI/0221/P015). After 36 h of incubation, the supernatant containing SARS-CoV-2S S protein pseudotyped VSV viruses (VSVppSARS-2S) was harvested and kept at −80°C for use in later investigations.

### Evaluation of VSVppSARS-2S entry into A549 cells through luciferase assay and fluorescence imaging

The effect of DSV treatment on the entry of VSVppSARS-2S pseudoviruses into A549 cells was assessed according to previously published protocols, luciferase assay was performed with a few minor modifications (Berger Rentsch and Zimmer, 2011; Hoffmann et al., 2020b) as per approval from the Institutional Biosafety Committee of Patanjali Research Institute (vide approval # IBSC/PRI/0221/P014). DSV pre-treated (1, 3, 10, and 30 μg/ml for 16 h) 70–80 percent confluent A549 cells were infected with VSVppSARS-2S [at a multiplicity of infection (m.o.i.) of 10] at 37°C for 6 h. Subsequently, after removing the medium, cell lysates were prepared in 50 μl of 1X cell culture lysis reagent. Firefly luciferase activity was measured in an Envision microplate reader (Perkin Elmer, United States) immediately after mixing 30 μl of the luciferase substrate, reconstituted in luciferase assay buffer, and 20 μl of the lysate. The extent of VSVppSARS-2S entry into the cells was reflected by the luciferase activity, which was expressed as “x-fold over background”. Uninfected and infected cells without DSV treatment were included as normal and disease controls, respectively. VSVppSARS-2S pseudovirus infected cells treated with 100 μM of camostat mesylate (Hoffmann et al., 2020b) were taken as the positive control. In a parallel experimental setup, A549 cells were infected with VSVppSARS-2S for 6 h (as mentioned above) for fluorescence imaging of EGFP-expressing cells. After fixing with 0.4% paraformaldehyde for 10 min at room temperature, the cells were washed thoroughly with sterile PBS. The cells were then imaged under bright and fluorescent (excitation at ∼445–495 nm) fields using an inverted microscope (Juli, GFP 10X-Smart Fluorescent Cell Analyser, NanoEntake, South Korea). The number of EGFP-expressing A549 cells were counted from images taken from six different fields for each sample.

### Evaluation of pseudotyped virus (VSVppSARS-2S)-induced cytokine response in A549 cells

50%–60% confluent A549 cells were infected with VSVppSARS-2S pseudoviruses. After 48 h of incubation, the quantities of pro-inflammatory cytokines, namely, IL-6, IL-1β, and TNF-α that were released in the supernatants of cultured A549 cells were estimated by using ELISA, as previously described (Balkrishna et al., 2021a).

### Secreted embryonic alkaline phosphatase based NF-κB/AP-1 reporter assay

THP1-Blue NF-κB reporter cells were used to monitor the level of NF-κB activation. Briefly, cells with a density of 5 × 10^5^/ml were seeded in a 96-well plate (Thermo Fisher Scientific, MA, United States) and co-treated with 10 ng/ml TNF-α (Catalogue #300-01A-50UG; Peprotech, Cranbury,NJ, United States) and DSV (1, 3,10 and 30 μg/ml) for 24 h. Post-incubation, SEAP was quantified using QUANTI-Blue Solution (InvivoGen, CA, United States) as per the manufacturer’s instructions. The optical density was read at 630 nm using an Envision (PerkinElmer, MA, United States) multimode plate reader.

### Statistical analysis

All experiments were carried out in triplicate at least three times independently and data were represented with mean ± standard error (SEM). Statistical significance of the variations observed between the mean of different groups and data points were evaluated through one-way ANOVA with Dunnett multiple comparison. GraphPad Prism software 8.0.2 (GraphPad Inc., San Diego, CA, United States) was used for statistical analyses and graphical representations of the quantitative data.

## Results

### DSV ameliorated poly I:C induced pro-inflammatory cytokine response in THP-1 cells at cyto-safe doses

**FIGURE 1:**
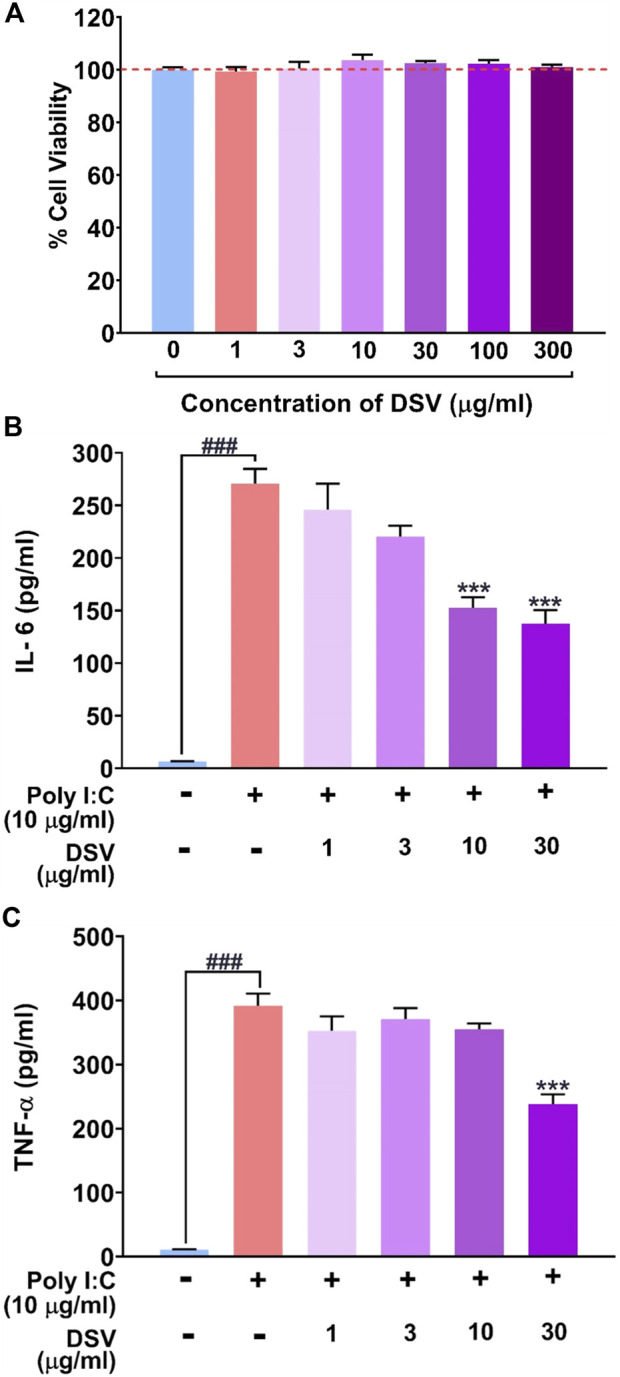
DSV attenuated Poly I:C induced pro-inflammatory cytokine response in THP-1 macrophage cells. **(A)** Cytotoxicity of DSV on THP-1 cells expressed as % cell viability. Similar cell viabilities in untreated and DSV treated cells are indicated with the broken red line. **(B,C)** THP-1 cells were pre-treated with DSV for 16h followed by its co-treatment with Poly I:C for 24 h and assayed for the secretion of cytokines, IL-6 **(B)** and TNF-α **(C)** using Enzyme-Linked Immunosorbent Assay (ELISA). Data on the levels of secreted cytokines are represented as mean ± SEM from three independent experiments. The statistical significance of the differences observed between the means was analysed through one-way ANOVA followed by Dunnett’s multiple comparison test and represented as ### for *p* < 0.001 when compared to the normal cells without DSV and Poly I:C treatment and as *** for *p* < 0.001 compared to poly I:C-induced cells without DSV treatment.

In the *in vitro* cytotoxicity assay, treatment of THP-1 cells with various concentrations of DSV (0–300 μg/ml) for 24 h revealed no significant effects on the cell viability even when the cells were exposed to the highest concentration used for the assay as evident in Figure 1A. Up to 300 μg/ml under *in vitro* conditions, DSV was considered to be cytosafe for THP-1 cells. A much lower range of DSV concentrations (1–30 μg/ml) was used for subsequent *in vitro* assays. Elicitation of pro-inflammatory cytokine levels in THP-1 macrophage cells as a host defence mechanism against infections is a common phenomenon. In the event of SARS-CoV-2 infection, immune cells can develop the CSS condition with hyper-expressed pro-inflammatory cytokines. This condition was mimicked *in vitro* by incubating THP-1 macrophage cells with an inducer such as Poly I:C. Polyinosinic:polycytidylic acid (Poly I:C) is a synthetic immunostimulant that mimics double-stranded RNA, a molecular pattern associated with viral infections and easily recognized by TLR3 of the host innate immune system. By mimicking viral infection, exposure to Poly I:C can stimulate virus-induced cytokine and interferon production in mammalian cells (Tamir et al., 2022). For this assay, 24 h of exposure to 10 μg/ml Poly I:C significantly raised the secreted levels of IL-6 (Figure 1B) and TNF-α (Figure 1C) in cell culture supernatants as compared to the negative control where the inducing agent, as well as DSV exposure, were absent. DSV was competent enough to reduce Poly I:C-induced IL-6 and TNF-α in a dose-dependent manner. Interestingly, 30 μg/ml DSV was shown to reduce Poly I:C-induced cytokine levels up to 50%.

### DSV interfered with human ACE 2 receptor and SARS-CoV-2 S protein interaction

**FIGURE 2:**
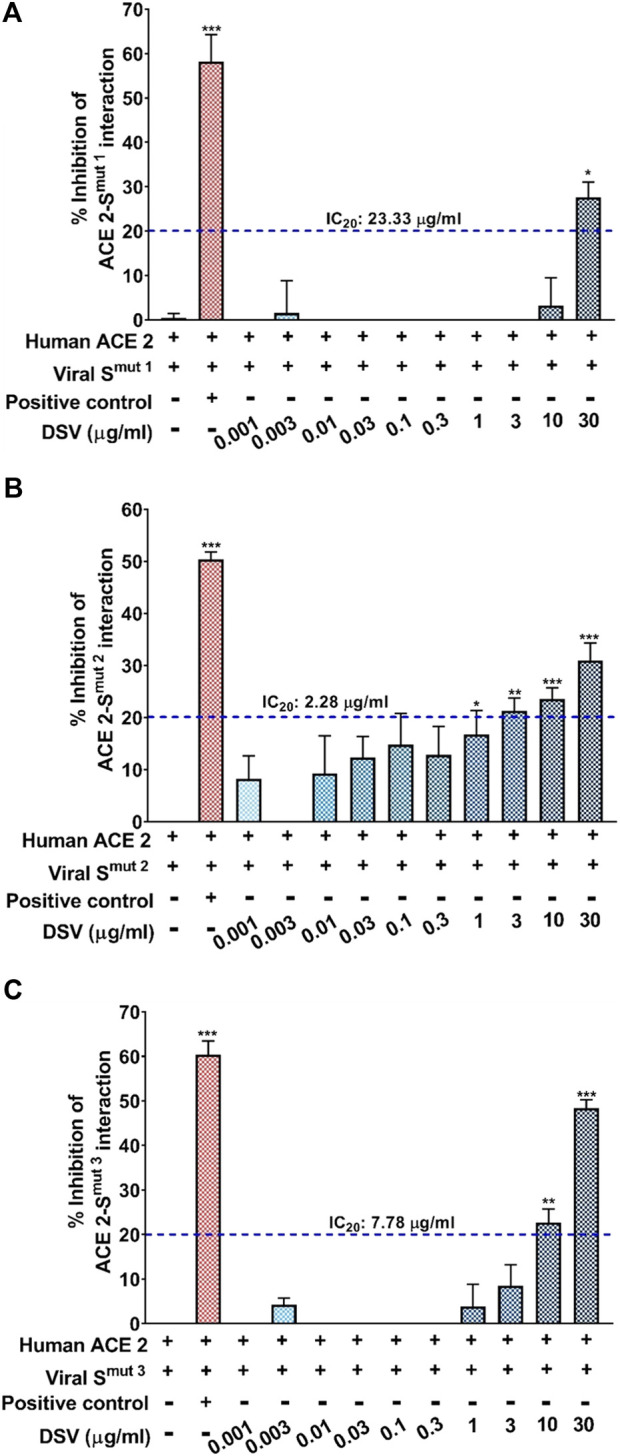
DSV inhibited interaction between human ACE 2 receptor and SARS-CoV-2 S proteins. **(A–C)** ELISA-based assay to quantify the dose-dependent effect of DSV on the interactions between human ACE 2 receptors and different types of SARS-CoV-2 S proteins, namely, S^mut1^ **(A)**, S^mut2^ **(B)** and S^mut3^ **(C)** and represented as percentage (%) inhibition relative to the extent of ACE 2-S protein interactions observed in the reaction mix without any inhibitor. Data represented as mean ± SEM from three independent experiments. The statistical significance of the observed differences in the means compared to the no inhibitor group [first column in each case from **(A–C)**] was analysed through one-way ANOVA followed by Dunnett’s multiple comparison test and represented as *, ** or *** depending on whether the calculated *p* value was <0.05, <0.01 or <0.001. IC_20_ concentrations of DSV against ACE 2 interaction with each variant of S protein were determined and indicated.

In light of the early findings from the previous experiment, it was intriguing to look closely at the molecular docking studies and experimental results obtained by independent research groups on the constituent herb/s or active phytocomponent/s present in the DSV formulation such as *Pistacia chinensis* subsp. *integerrima* (Kumar Paul et al., 2022). Glycyrrhizin/liquorice extract (Luo et al., 2020) and coumarins (Abdelmohsen et al., 2021) have been shown to block molecular interaction between S-protein and hACE 2. These previous converging reports act as a foundation for establishing DSV as a potent anti-SARS-CoV-2 agent. Therefore, an ELISA-based biochemical assay was conducted to decipher the effect of DSV in restricting the interaction between hACE 2 and viral S protein. For this assay, we have tested the interaction of three different viral SARS-CoV-2 S protein mutants namely, S^mut1^ (the one identified at the beginning of the pandemic), S^mut2^ (W436R) and S^mut3^ (D614G), immobilized on an ELISA plate with biotinylated purified human ACE 2 protein. The pharmacological inhibitor of ACE 2-S protein interaction provided in the kit served as a positive control, whereas the reaction mixture without any control or experimental inhibitor served as the negative control yielding complete ACE 2-S protein/s interaction. Consequently, the inhibition compared to the positive control caused by different concentrations of DSV was calculated. As evident from (Figures 2A–C), DSV exerted a dose-dependent inhibitory effect on the interactions between ACE 2 and all variants of S proteins, although there is some variation in the strength of the inhibition across all S protein mutants. The experimental setup found a 30%–50% DSV-driven inhibition of the ACE 2-S protein interaction. To compare the inhibitory effect of DSV among these S protein mutant variants, the IC_20_ values of DSV against each variant were calculated. They were found to be 23.33, 2.28, and 7.78 μg/ml for viral S^mut1^, S^mut2^, and S^mut3^ respectively. DSV exhibited a more effective inhibitory potency when S^mut2^ interacted with the ACE 2 receptor. These findings unambiguously depicted the potency of DSV in preventing viral S proteins from communicating with human ACE 2 receptors at the molecular level.

### DSV moderated levels of viral S protein-induced inflammatory cytokines in A549 cells

**FIGURE 3:**
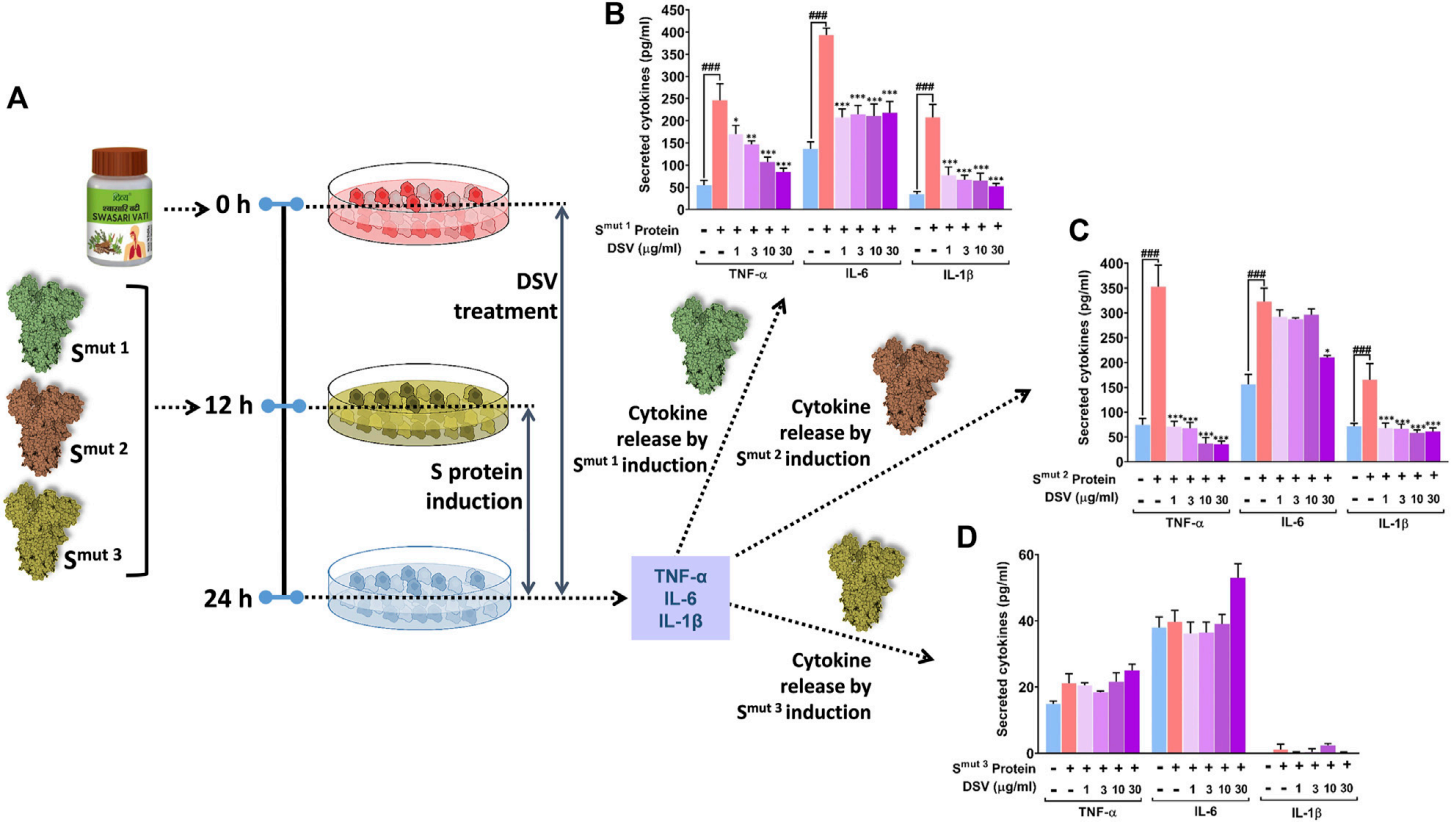
DSV attenuated viral S protein-induced cytokine activation in A549 cells. **(A)** Schematic representation of the experimental plan. **(B–D)** Activation of pro-inflammatory cytokines in response to SARS-CoV-2 S protein variants, S^mut1^ **(B)**, S^mut2^ **(C)** and S^mut3^ **(D)** in alveolar epithelial A549 cells and its dose-dependent amelioration upon DSV treatment were measured by ELISA. Observations are depicted as levels of secreted cytokines. Data represented as mean ± SEM from three independent experiments. The statistical significance of the differences observed between the means was analysed through one-way ANOVA followed by Dunnett’s multiple comparison test and represented as ### for *p* < 0.001 when compared to normal cells without DSV treatment and exposure to S protein and as *, **, and *** for *p* < 0.05, 0.01, and 0.001, respectively, when compared to cells exposed to S protein induction but not treated with DSV.

ACE 2 receptor expression makes the alveolar type II (AT- II) A549 epithelial cells a favourable target for SARS-CoV-2 infection. Therefore, the aforementioned cell line is chosen for testing our formulation *in vitro* studies (Foster et al., 1998). In continuation with our earlier observations where DSV downregulated Poly I:C induced cytokines in THP-1 and restricted the ACE 2-S protein interaction, it was further conceived that the DSV could downregulate S-protein-induced cytokines in A549. To assess this premise, A549 lung epithelial cells were pre-treated with DSV at doses of 1, 3, 10 and 30 μg/ml for 12 h, and subsequently, the activation and release of three immune-reactive pro-inflammatory cytokines (TNF-α, IL-6, and IL-1β) in response to the introduction of viral S-protein variants (used earlier) were examined (Figure 3A). Cells unexposed to S proteins and untreated with DSV were taken as normal control, while those incubated with S-proteins but not treated with DSV were included as disease control. S^mut3^ (Figure 3D) does not have the same effect on any of the measured cytokines as S^mut1^ (Figure 3B) or S^mut2^ (Figure 3C), which showed significantly high levels of all three immune-reactive cytokines in the disease control. Interestingly, these cytokines were decreased in cells treated with a gradient of DSV at increasing concentrations. However, only a few instances of dosage dependence were observed. As shown in (Figure 3B), levels of both TNF-α and IL-1β in the cells were elevated by about five folds in response to S^mut1^. These cytokine levels were restored almost to normal by DSV treatment in a dose-dependent manner. Notably, a steeper fall in the S^mut1^-induced cytokines level after DSV-treatment on A549 cells is observed for IL-1β compared to TNF-α. As an illustration, 1 μg/ml of DSV treatment lowered S^mut1^-induced TNF-α and IL-1β on A549 cells by ∼1.4 and ∼2.5 folds respectively.

IL-6 in A549 cells was found to increase by about 3.5-folds in response to S^mut1^. DSV treatment significantly reduces this elevated cytokine level to nearly half compared to the cytokine level obtained for disease control. However, no dose-dependent effect of DSV was observed in the case of this mutant. Compared to control groups, TNF-α, IL-6 and IL-1 levels elevated almost 5, 2.2, and 2.5-fold in the disease control groups of S^mut2^ protein-treated cells, respectively (Figure 3C). DSV demonstrated high resilience to these elevated cytokine levels, effectively restoring TNF-α, and IL-1β to normal control at a dose as low as 1 μg/ml, without any apparent dose-dependency. Treatment with 30 μg/ml DSV is required for a significant reduction of IL-6 that brings down its level closer to the normal control. While analysing S^mut3^ protein-treated cells, we did not find any significant change in any of the cytokines studied, when compared to normal control groups (Figure 3D). Besides, a negligible amount of IL-1β levels were detected in the case of S^mut3^ variant. Since no induction in the cytokine levels was observed in S^mut3^ treated cells, the discernible effect of DSV treatment was unapparent. In summary, it is evident that all purified S protein mutant variants, except S^mut3^, elicited *in vitro* cytokine response in pulmonary epithelial cells and DSV was found to mitigate that response mostly restoring the cytokine levels to normalcy with varying efficiency based on the type of mutation that exists in the viral S protein.

### DSV inhibited VSVppSARS-2S entry into alveolar epithelial cells

**FIGURE 4:**
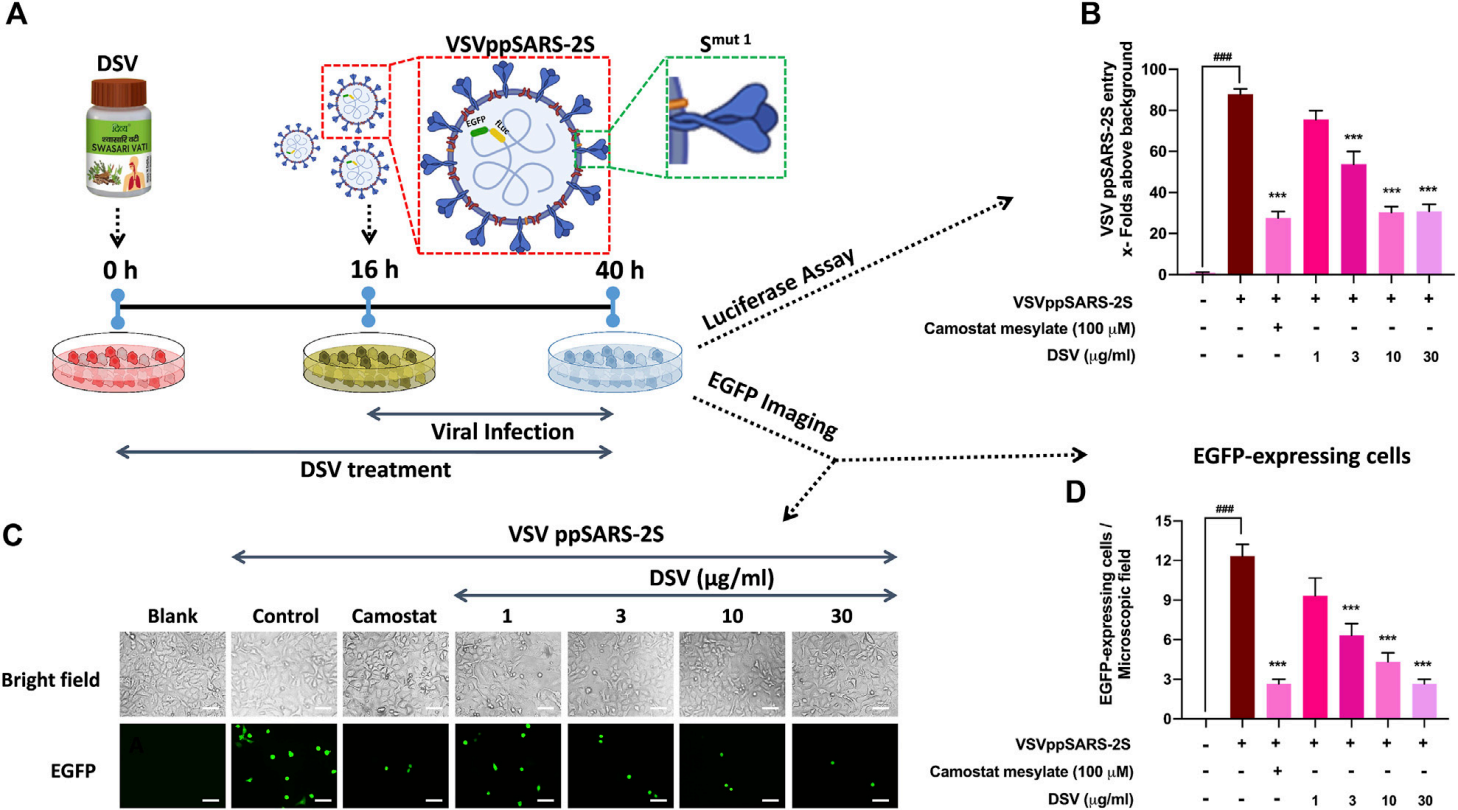
DSV inhibited entry of VSVppSARS-2S in alveolar epithelial cells. **(A)** Schematic representation of the experimental plan. **(B)** VSVppSARS-2S entry into the transduced A549 cells and the effect thereupon due to treatment with DSV and positive control Camostat mesylate, assessed through luciferase assay, were represented as fold change in luminescence units relative to the normal cells, taken as background. **(C)** Representative fluorescent micrographs of uninfected cells, VSVppSARS-2S virus-infected cells, infected cells treated with positive control Camostat mesylate and different concentration of DSV. *Scale bar: 50 μm* **(D)** Average number of EGFP positive cells in uninfected A549 cells, virus-infected cells, infected cells treated with positive control Camostat mesylate and DSV, determined from six different fields. Data represented as mean ± SEM from three independent experiments. The statistical significance of the differences observed between the means was analysed through one-way ANOVA followed by Dunnett’s multiple comparison test and represented as ### for *p* < 0.001 when compared to normal cells without DSV treatment and exposure to S protein and as *** for *p* < 0.001, when compared to cells infected with VSVppSARS-2S virus but not treated with DSV.

DSV effectively moderated the S protein-mediated rise in pro-inflammatory cytokines. Therefore, it can be conceived as a rational approach to study the effect of DSV on hindering the interaction of S protein expressing SARS-CoV-2 virus with A549 cells expressing hACE 2 receptors. To eliminate the need for specialized containment facilities to work on SARS-CoV-2, replication-defective, S protein pseudotyped infective virus particles were generated (Whitt, 2010). Host A549 cells were pre-treated with DSV at different concentrations (1, 3, 10, and 30 μg/ml) for 16 h before exposing them to VSVpp2S viruses for another 24 h in the presence of DSV. Thereafter, viral entry was assessed through luciferase assay and fluorescence imaging (Figure 4A). Untreated cells that had been pseudovirus transduced and uninfected cells that had not been transduced were used as disease and normal controls, respectively. The expression of reporter genes that have been stably integrated into the VSVppSARS-2S genome by infected host cells allowed the measurement of the amount of VSVppSARS-2S that entered the lung epithelial A549 cells. These reporters include the firefly luciferase gene (FLuc) and EGFP, which produced luciferase activity and green fluorescence in the infected cells, respectively. As demonstrated in (Figure 4B), compared to uninfected cells (considered as background), pseudovirus-transduced cells displayed about 80 times as much virus entry. An almost 3.5-fold decline in virus entry was observed in the infected cells treated with camostat mesylate, a serine protease inhibitor effective against TMPRSS 2 (Hoffmann et al., 2020b). Remarkably, a significant dose-dependent reduction of the virus entry in transduced cells, as compared to untreated cells, was observed upon DSV (1, 3, 10 and 30 μg/ml) pre-treatment. The reduction in viral entry into the host cell due to DSV treatment was comparable to the observed camostat mesylate effect. The results obtained by luciferase assay corroborated with the microscopic analysis of EGFP-expressing cells (Figure 4C) and were subsequently represented in quantifiable form (Figure 4D). Altogether, the results acquired through these assays establish the ability of DSV to prevent the entry of SARS-CoV-2 into host cells.

### DSV subdued VSVppSARS-2S-induced cytokine upsurge in alveolar epithelial cells

**FIGURE 5:**
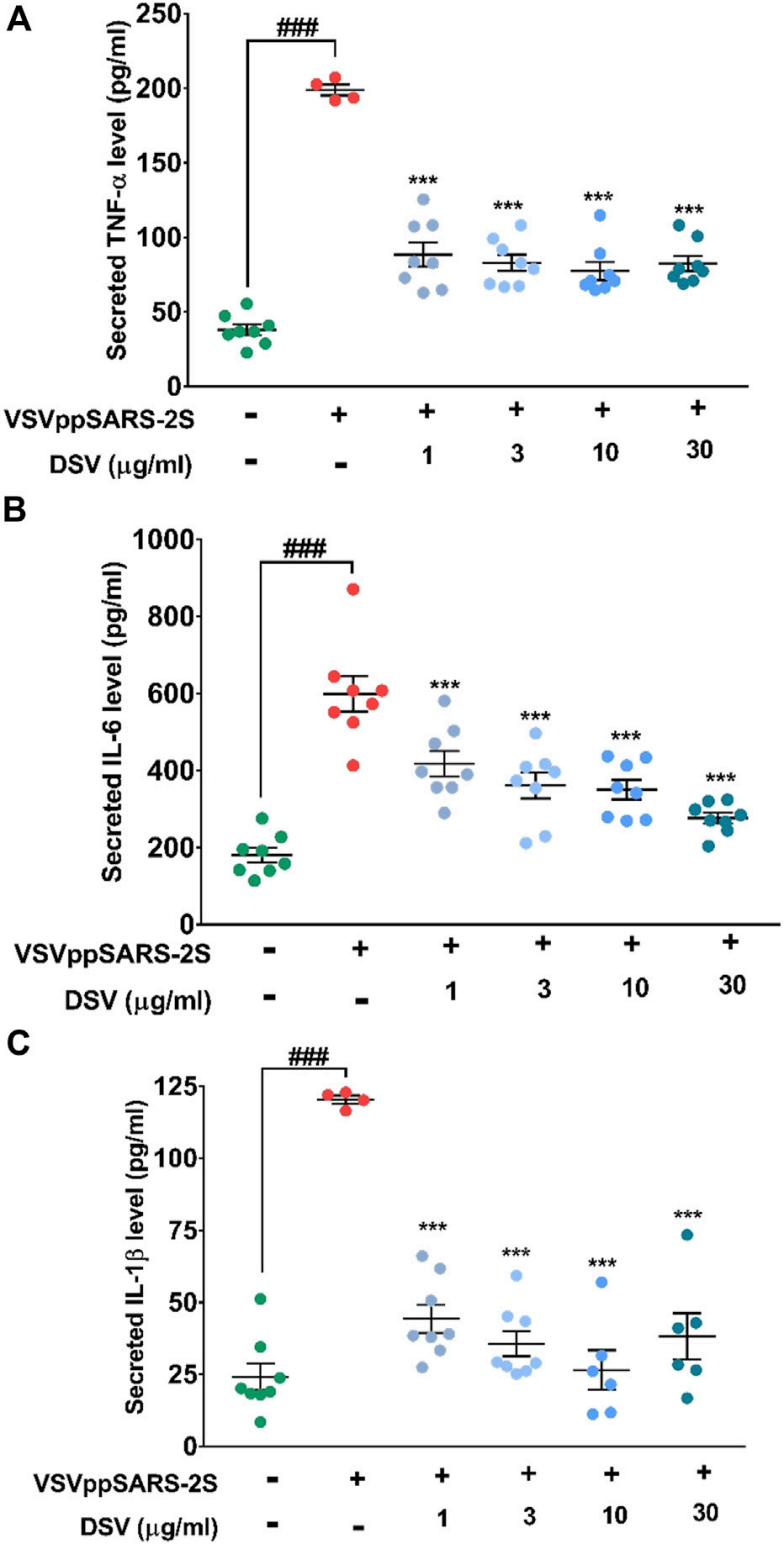
DSV reduced pseudovirus elicited cytokine response in A549 cells. **(A–C)** Cells were pre-treated with DSV followed by co-treatment with pseudovirus infection with VSVppSARS-2S for 24 h and assayed for the secretion of pro-inflammatory cytokines, TNF-α **(A)**, IL-6 **(B)** and IL-1β **(C)** by ELISA. Measured levels show the evoked cytokine responses and their reduction upon treatment with DSV. Results from three independent experiments were represented as mean ± SEM levels of secreted cytokines. The statistical significance of the differences observed between the means was analysed through one-way ANOVA and represented as ### for *p* < 0.001 when compared to non-transduced cells and as *** for *p* < 0.001 when compared to untreated virus-transduced cells.

Previous results indicated efficient management of S protein-evoked cytokines by DSV and its ability to inhibit viral entry. SARS-CoV-2 is known to trigger the immune system and cause CSS (Moore and June, 2020). Owing to the presence of functional S protein in a replication-defective, non-pathogenic VSVppSARS-2S mimics infection mechanism and pathophysiology of an infective SARS-CoV-2 virus that involves an increase in inflammatory cytokine (cytokine storm) secretion (Fara et al., 2020). Thus, ELISA was used to evaluate the amounts of immune-reactive pro-inflammatory cytokines secreted by VSVppSARS-2S-transduced A549 cells with and without DSV treatment. Uninfected cells without DSV treatment were used as normal control, whereas VSVppSARS-2S transduced A549 cells were used as disease control (Figures 5A–C). A549 cells infected with the VSVppSARS-2S virus underwent 4, 3, and 5-fold increases in TNF-α, IL-6, and IL-1β levels compared to the normal control, respectively. Pre-treatment and co-treatment of the infected cells with varying concentrations of DSV (1, 3, 10, and 30 μg/ml) dramatically lowered, TNF-α, IL-6, and IL-1β levels by 50, 33 and 60%, respectively, compared to the disease control. The reduction in the levels of secreted cytokines was noticeable with 1 μg/ml of DSV treatment, and it nearly reached the usual normal control level (uninfected) for all tested cytokines with the highest DSV concentration (30 μg/ml) taken for the assay. A decrease in IL-6 level in response to DSV treatment exhibited a dose-dependent trend. Overall, these observations ascertained the potential of DSV to counteract viral infection-induced cytokine release syndrome.

### DSV attenuated TNF-α- induced transcriptional activity of NF-κB

**FIGURE 6:**
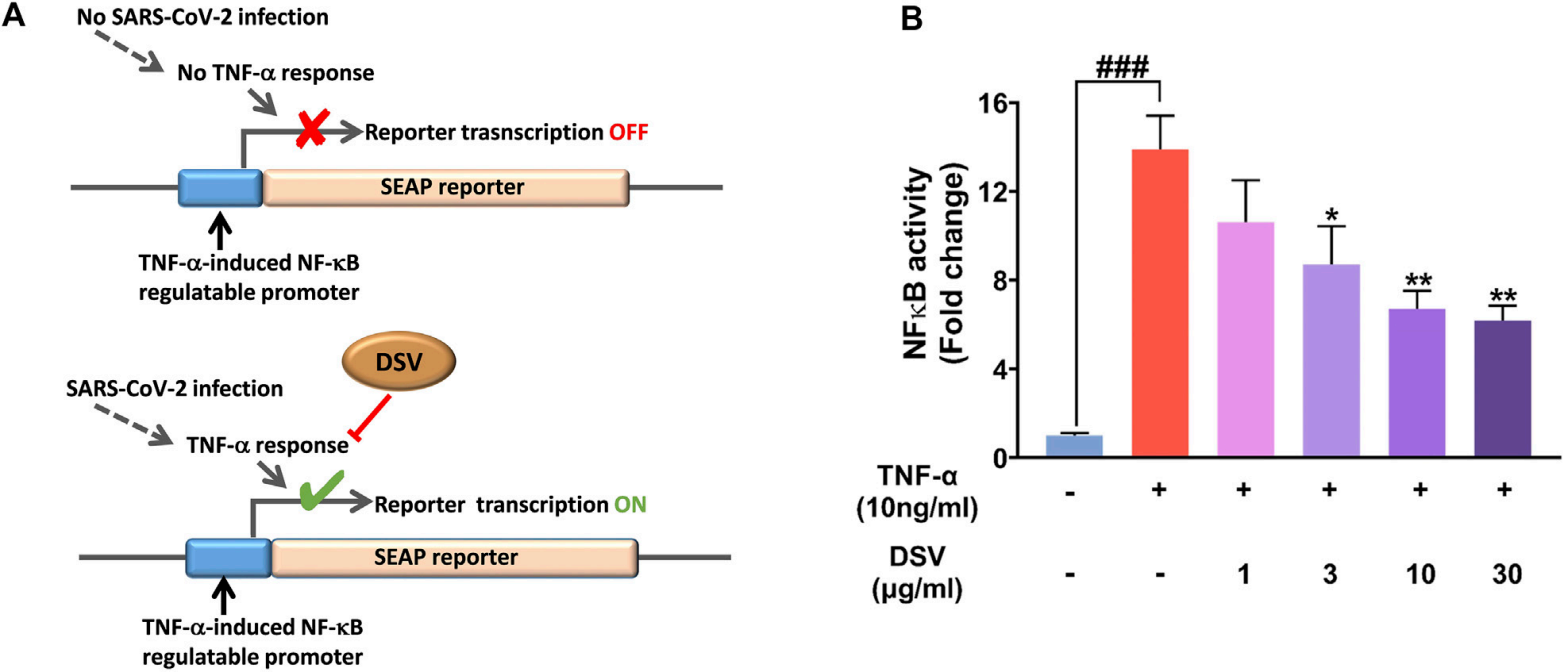
DSV inhibited TNF-α induced NF-κB transcriptional activity. **(A)** Schematic representation of the experimental rationale. **(B)** THP1 Blue cells stably expressing the NF-κB inducible SEAP reporter gene were pre-treated with various doses of DSV followed by treatment with TNF-α and SEAP activity measured after 24 h. Results from three independent experiments were represented as mean ± SEM levels of secreted cytokines. The statistical significance of the differences observed between the means was analysed through one-way ANOVA and represented as ### for *p* < 0.001 when compared to normal cells without DSV and TNF-α treatment and as * and ** for *p* < 0.05 and 0.01, respectively, when compared to cells without DSV treatment but exposed to TNF-α.

A significant pathogenic trait of the SARS-CoV-2 infection is the production of a cytokine storm characterized by a massive increase in pro-inflammatory cytokines (Coperchini et al., 2020). NF-κB is the master regulator involved in lung inflammation (Barnes, 2006), and TNF-α is a very effective inducer of the NF-κB signalling pathway (Hayden and Ghosh, 2014). TNF-α-induced NF-κB signaling pathway associated with neuroinflammation (CIPN) is well known (Fumagalli et al., 2020; Zhang P. et al., 2020). Besides, the NF-κB pathway has been identified as a potential therapeutic target in the treatment of COVID-19 (Kircheis et al., 2020). Our earlier observations showing the pro-inflammatory cytokine modulating effect of DSV, together with these converging evidence, encouraged us to assess the effect of DSV on TNF-α-mediated NF-κB induction. Accordingly, TNF-α-induced activation of NF-κB promoter was assessed through transactivation assay using THP1-Blue™ NF- κB reporter system. THP1-Blue™ is a stable cell line engineered to express SEAP enzyme from an NF-κB regulatable promoter. The addition of TNF-α triggers NF-κB mediated SEAP expression, which is quantifiable through an enzymatic reaction. This quantification indirectly reflected the treatment-mediated modulation of TNF-α-facilitated NF-κB induction (Figure 6A). Cells exposed to TNF-α with no DSV treatment are the disease control, while cells without TNF-α and DSV treatment act as normal control where NF-κB transcriptional activity is absent. The addition of 10 ng/ml TNF-α induced an ∼14 -fold increase in NF-κB promoter induction activity compared to the normal control, while co-treatment of these reporter cells with DSV significantly inhibited the TNF-α induced NF-κB transcriptional activation in a dose-dependent manner (Figure 6B). At the highest DSV concentration (30 μg/ml) used for the assay, the TNF-α-induced NF-κB transcriptional activation falls to nearly 50% of the untreated disease control. Therefore, taken together, these observations corroborate the anti-inflammatory properties of DSV and demonstrate the potential of DSV in moderating the undesirable cytokine-storm (TNF-α)-induced NF-κB induction. These observations strongly implicate that DSV is not only capable of countering the upsurge of cytokines during SARS-CoV-2 infection, but also has the potential to prevent the inflammatory response from going into an overdrive by targeting the NF-κB pathway.

## Discussion

SARS-CoV-2 infection is contagious, with a high mortality rate. Catastrophic prognosis includes suppressed immunity, cytokine storm, organ failure and susceptibility to pneumonia (Ragab et al., 2020). During the crisis, a concerted effort by the global scientific community was made towards repurposing certain already available medications, such as umifenovir, camostat mesylate, hydroxychloroquine, soluble recombinant hACE 2 that induced a drop in the levels of S protein bound to the cell membrane mACE 2, S protein-specific monoclonal antibodies or fusion inhibitors like arbidol, immunosuppressants (like, dexamethasone, IL6 inhibitory monoclonal antibody tocilizumab, IL-1β receptor antagonist anakinra and sarilumab) (Hu et al., 2021), viral replication inhibitors (remdesivir, favilavir, ribavirin, lopinavir, and ritonavir) (Beigel et al., 2020). Combinatorial treatment regimens were also considered keeping the pharmacokinetics of the medicines and the patient’s risk profile in perspective (Felsenstein et al., 2020). However, the efficacy of none of these treatment options was found to be optimal against COVID-19, let alone the severe side effects, especially for immunocompromised cancer and diabetic patients (Di Lorenzo et al., 2020). During the initial phase of the pandemic, several *in silico* studies reported potentially effective metabolites against SARS-CoV-2 infection (Balkrishna et al., 2021a; Balkrishna et al., 2021b; Kumar V. et al., 2021; Kumar et al., 2022). Several herbal plants target immune responses by reducing cytokine levels (Li S. et al., 2021). During the COVID-19 pandemic, the knowledge base of traditional systems of medicines from India and China proved quite effective. Traditional Chinese Medicine (TCM) has been successfully applied to combat the SARS-CoV-2 pandemic (Yang Y. et al., 2020). In fact, as emergency measures, governments in India and China approved traditional medicines (based on medicinal herbs and natural products) as supporting measures to manage COVID-19 during the peak of the pandemic. Traditional medicinal concoctions of natural products contain bioactive components in a well-defined functional matrix capable of multi-targeting a disease etiology. The concept of poly-herbalism in formulating medicinal concoctions to achieve greater therapeutic efficacy with reduced toxicity is highlighted in Ayurvedic literature called *Sarangdhar Samhita* (Parasuraman et al., 2014). Clinical trials of anti-viral drugs such as hydroxychloroquine (Boulware et al., 2020) and remdesivir (Beigel et al., 2020) yielded ineffective results against the SARS-CoV-2 virus. In contrast, promising SARS-CoV-2 clearance and fast recovery with no adverse effects were observed for a placebo-controlled pilot-scale pre-clinical trial carried out with ayurvedic treatment regime on COVID-19 positive patients (Devpura et al., 2021). DSV is a classical Ayurvedic formulation that has been used to treat ailments of the respiratory system, including asthma, for thousands of years. This, along with the ability of DSV to ameliorate the pathology induced by the SARS-CoV-2 S protein in zebrafish (Balkrishna et al., 2020c) warranted a detailed study to gain mechanistic insight into the mode of action of this formulation as an anti-SARS-CoV-2 agent. Therefore, the current study was designed to evaluate the anti-SARS-CoV-2 activity of DSV by monitoring its effect on viral-host interaction levels.

DSV is a herbo-mineral Ayurvedic formulation whose individual metabolites are reported to have anti-viral, anti-inflammatory and immunomodulatory properties. The anti-SARS-CoV-2 property was also shown by another major DSV metabolite, glycyrrhizin, a triterpene saponin that has a long history of medical use. It can not only bind ACE 2 to prevent SARS-CoV-2 infection (Luo et al., 2020), but can also inhibit SARS-CoV-2 replication by blocking COVID-19 main protease M^pro^ (Van De Sand et al., 2021). Besides, glycyrrhizin is effective against many other viruses such as hepatitis, dengue and influenza (Bailly and Vergoten, 2020). Glabridin is capable of binding at the active site of M^pro^, suggesting an enzyme inhibitory effect (Srivastava et al., 2022). 6-Gingerol and eugenol, two other metabolites present in DSV, are shown to have a high binding affinity for M^pro^ active sites. Apart from SARS-CoV-2, 6-Gingerol was reported to exhibit potent anti-viral activity against chikungunya virus (CHIKV) infection of human hepatocyte HepG2 cells (Hayati et al., 2021) and human respiratory syncytial virus (HRSV) in respiratory upper and lower tract cell lines, HEp-2 and A549, respectively (Chang et al., 2013; Rathinavel et al., 2020; Chandra Manivannan et al., 2022). Eugenol was found to bind SARS-CoV-2 S1 protein to inhibit the host-pathogen interaction between human ACE 2 receptor and viral S protein (Paidi et al., 2021). Gallic and ellagic acids also showed potential binding with RNA-dependent RNA polymerase of SARS-CoV-2 in molecular docking studies (El Sohaimy et al., 2021). Besides, gallic and ellagic acids, together with protocatechuic acid, were also found to target 3CL^pro^ of the SARS-CoV-2 virus (Arokiyaraj et al., 2020). Molecular docking studies with coumarin, also one of the identified marker metabolites present in DSV, revealed good docking scores indicating strong binding with RBD of S-protein, which could inhibit the viral attachment and entry into human host cells (Abdelmohsen et al., 2021). It is the major bioactive molecule of *Cressa cretica*, one of the nine medicinal herbs present in DSV that is effective as an anti-viral agent against a wide range of viruses such as influenza, dengue, chikungunya and enterovirus. Coumarin is known to inhibit NF-κB signaling, which is crucial for viral entry and replication (Mishra et al., 2020). *In silico* studies by Kumar G. et al. (2021) had shown that piperine from *Piper nigrum* and curcumin from *Curcuma longa* effectively bind to the ACE 2 receptors of the host cell. Similarly, a study by Pandey et al. (2020) demonstrated that the metabolites present in *Piper nigrum, Syzygium aromaticum and Zingiber officinale* could target the main protease of SARS-CoV-2. Methyl gallate (Correa et al., 2020) and cinnamic acid (Yakhchali et al., 2021) are very good anti-inflammatory agents with strong immune-modulatory potentials, which are likely to attune the over-driven inflammatory responses during SARS-CoV-2 infection. *Pistacia chinensis* subsp*. integerrima,* one of the most abundant herbal components of DSV, contains 28-dimethyl-β-amyrone, 24-Noroleana-3,12-diene, and stigmasterol. These metabolites, although not detected in DSV formulations *per se*, have demonstrated strong binding with S protein RBD of SARS-CoV-2, forming stable complexes, thereby attenuating its interaction with the host cell (Kumar Paul et al., 2022).

Our study focused on studying the effect of DSV in ameliorating viral infection-induced pro-inflammatory cytokines upsurge. The inflammatory response may occur due to an infestation of pulmonary pathogens such as *Mycoplasma pneumoniae* (Yang et al., 2002), bacterial endotoxin like liposaccharide (Crestani et al., 1994; Lin et al., 2020) or viruses such as H1N1 infection (Wang et al., 2015) and respiratory syncytial virus (Levitz et al., 2012). Several previous reports have indicated a sudden violent outburst of systemic cytokine levels in SARS-CoV-2 infected individuals, instigating a cascade of detrimental autoimmune consequences known as CSS (Mehta et al., 2020). The key perpetrating pro-inflammatory cytokines which were targeted to manage the symptoms of COVID-19 are IL6 and TNF-α (Costela-Ruiz et al., 2020; Moore and June, 2020). The primary source of pathogenic IL-6 induced by SARS-CoV-2 infection appears to be myeloid cells (Gubernatorova et al., 2020). Moreover, several inflammatory cytokines associated with NF-κB signaling pathways (Su et al., 2021), JAK/STAT and MAPK/ERK are also involved in the progression of the cytokine storm (Dosch et al., 2009). The primary goal of an anti-COVID therapeutic strategy is to alleviate excess production of cytokines. According to a report, immunosuppressant compounds derived from plant sources like curcumin, luteolin, piperine, and resveratrol are known to inhibit the production and release of pro-inflammatory cytokines and chemokines (Peter et al., 2020). Medicinal plants present in DSV, like *P. chinensis* subsp*. integerrima* reportedly subdued the expressions of inflammatory molecules, TNF-α, IL-4, and IL-5 in the mouse model of ovalbumin (OVA) induced allergic asthma, thereby alleviating pulmonary edema. This *in vivo* immunomodulatory activity can be helpful in the treatment of asthma and cough (Rana et al., 2016). Consistent with this observation, Divya-Swasari-Ras (DSR) demonstrated suppression of pro-inflammatory cytokine response in the mouse model with ovalbumin (OVA)-induced allergic asthma (Balkrishna et al., 2020a). Furthermore, *P. chinensis* subsp*. integerrima* exhibited high anti-oxidant activity. Glycyrrhizin, a marker metabolite of DSV, displayed diverse pharmacological properties, including anti-cancerous, anti-thrombotic, anti-mutagenic, anti-ulcerogenic (Isbrucker and Burdock, 2006) anti-microbial, anti-parasitic, anti-allergenic, anti-tussive (Hasan et al., 2021), hepatoprotective, anti-oxidative, and anti-inflammatory properties attributed to its inhibitory effect on NF-κB, that helps in abating pro-inflammatory cytokines. Reactive oxygen species (ROS) and Respiratory Distress Syndrome (RDS) are induced by viral infections (Ghosh et al., 2011; El-Saber Batiha et al., 2020). Other metabolites present in DSV, like piperine, exhibit a strong anti-oxidant and immunomodulatory effects (Miryan et al., 2021). In addition to herbal components, DSV also contains seven different *bhasmas* that maintain optimal alkalinity and potential anti-inflammatory activity (Balkrishna et al., 2020c). It was alluring to assess the vast computational discoveries and numerous experimental evidence indicating the broad spectrum of anti-viral capabilities of herbal constituents of DSV that complements our current endeavour.

The ability of DSV to inhibit hACE-2-S protein interactions was evaluated using an ELISA-based protein-protein interaction assay. For this, a panel of three different SARS-CoV-2 S protein mutants was used, namely, viral S^mut1^ (one identified at the beginning of the pandemic), viral S^mut2^ (W436R) and viral S^mut3^ (D 614G). A global sampling of SARS-CoV-2 indicates rapid mutations in spike protein amino acid sequences, which presumably allow the virus to gain an evolutionary advantage of greater infectivity by evading host anti-viral responses and geographical prevalence by improved transmissibility. The major strain of SARS-CoV-2 predominant in global circulation during pandemic has the S^mut3^ mutation. This mutation of the S protein has implications in attaining greater viral infectivity, as PCR-based studies revealed that people infected with this variant of SARS-CoV-2 had higher viral loads in the upper respiratory tract (Korber et al., 2020). The greater incorporation of S protein into the virions during packaging accounts for their improved infectivity and transmissivity (Zhang L. et al., 2020). S protein-hACE 2 binding affinity and the severity of disease caused by S^mut2^ mutation is comparable to other mutant variants of SARS-CoV-2. Apparently, this fact is noticeable as a nearly identical percentage of the inhibitory effect caused by positive control (chemical inhibitor provided with the Elisa kit) during ACE 2-S protein interaction studies with all three S protein mutants. However, the inhibitory effect exerted by DSV on hACE 2–S^mut3^ is comparatively more than on hACE 2–S^mut1^. This reflects the ability of DSV to curb the infection caused by a comparatively more infectious/transmissible stain of SARS-CoV-2. Moreover, there was no increase in the levels of released pro-inflammatory cytokines, namely IL-6, TNF-α, and IL-1β in A549 cells after S^mut3^ protein exposure confirming the clinical evidence that there is no correlation between the illness severity with this mutation (Zhang L. et al., 2020). RBD of S protein plays a crucial role in viral attachment, entry and subsequent infection. Mutations in specific residues of RBD known as “hot-spot” under high positive selection pressure develop mutant strains that displayed enhanced ACE 2 affinity. S^mut2^ has a RBD hot-spot mutation where incorporation of positively charged arginine enabled S protein to interact strongly with highly negatively charged ACE 2 surface. There are also cold-spot residues where point mutation can result in decreased interactions (Padhi and Tripathi, 2020; Ou et al., 2021). Owing to hot-spot mutation, the ACE 2–S^mut2^ interaction is stronger than the ACE 2–S^mut1^ complex. It is noticeable that DSV inhibited hACE 2–S^mut2^ interactions much more efficiently with the least IC_20_ value compared to hACE 2–S^mut1^ and hACE 2–S^mut3^. Our observations also indicate that S^mut2^ protein-induced elicitation of secreted pro cytokine response in A549 cells was conspicuous and virtually equivalent to that seen with S^mut1^ protein, and it gets effectively attenuated by DSV.

SARS-CoV-2 S protein pseudotyped vesicular stomatitis virus (VSV) infection of A549 cells resulted in the expression of the reporter gene (luciferase and EGFP) as well as elicited cytokine response. DSV successfully quelled both of them. These findings are explained collectively by the potency of DSV to excellently restrain the viral entry into the host cell. The ideal model system to examine SARS-CoV-2 entry and the associated inflammatory responses in human alveolar epithelial A549 cells as SARS-CoV-2 infection mostly targets human lung cells that abundantly express ACE 2 protein, a host cell receptor protein playing a vital role in viral attachment and entry. On stimulation by SARS-CoV-2 S proteins, A549 cells also produce a traceable amount of cytokine response. The maximum bioavailability of DSV under *in-vitro* conditions served as the foundation for our results. However, the effectiveness of an active molecule under *in vivo* conditions is often determined by its route of administration, blood concentration and binding to plasma protein and tissue (Mueller et al., 2004). Regarding pharmacokinetics, the *in vitro* experimental setup cannot be directly compared with *in vivo* circumstances. Thus, it is a common practice to employ drugs at concentrations exceeding the *in vitro* effective concentrations to obtain measurable activity under *in vivo* conditions. In the case of DSV, a pre-clinical study in zebrafish exhibiting symptoms of COVID-19 induced by SARS-CoV-2 S protein already established the pharmacological efficacy of this formulation (Balkrishna et al., 2020c). Nonetheless, in the current study, the *in vitro* pseudovirus and cell-based assays provided a platform to comprehend the mode of action of DSV as an inhibitor of SARS-CoV-2 entrance into host cells.

Our findings demonstrated that DSV blocks the interaction of viral S protein with ACE 2, thereby downregulating the virus infection-induced pro-inflammatory cytokines (IL-6), (IL-1β), and (TNF-α) which are highly elevated instigating the innate anti-viral response. Previous reports from our group had also validated the efficacy of DSV under *in vivo* experimental design where DSV attenuated pro-inflammatory cytokine response in an induced allergic asthma mouse model (Balkrishna et al., 2020b) and rescued humanized zebrafish (xenotransplanted with human alveolar epithelial A549 cells) from SARS-CoV-2 S-protein-induced pathologies (Balkrishna et al., 2020a). Mounting cytokines in response to viral infection influences each other and triggers other inflammatory pathways, such as NF-κB signaling. TNF-α which is also a major transcriptional regulator, is known to mediate the cytokine response through the action of NF-κB, a downstream effector. We found that TNF-α-mediated NF-κB induction could be moderated in a dose-dependent manner by DSV treatment. This indicated that DSV is endowed with immunomodulatory properties and helps in subduing the detrimental rise in inflammation.

Through our current research findings, we are successful in deciphering the potent anti-SARS-CoV-2 activity of DSV, particularly in terms of blocking the interaction of viral S protein with human ACE 2 receptor, preventing viral entry into the cells and ameliorating the associated immunogenic responses, thereby minimizing transition of innate to adaptive immune response. The likelihood of the adaptive reaction being overdriven is also diminished. This could ultimately reduce the chances of organ failure caused by anti-nucleoprotein antibodies and catastrophic disease consequences. All these can be attributed to the richness of DSV in metabolites that collectively display desirable positive traits.

## Conclusion

The current study has provided the mechanistic insight into the anti-viral activity of the herbo-mineral formulation Divya-Swasari-Vati (DSV) against SARS-CoV-2. Interaction between SARS-CoV-2 spike (S) protein and human host ACE 2 receptor is a prime requirement of COVID-19 infection, and concomitant severe inflammatory responses. Our observations from this study revealed that DSV inhibited this biomolecular interaction between viral S protein of different SARS-CoV-2 strains and host ACE 2 receptor, thereby inhibiting the viral entry into the human host lung epithelial A549 cells. DSV also curbed the immunogenic cytokine response mounted during SARS-CoV-2 infection of A549 cells *in vitro*, implicating its potential to attenuate the overdriven cytokine response, associated with severe COVID-19 infection. DSV moderated TNF-α-induced transcriptional activation of NF-κB signaling. The observed anti-SARS-CoV-2 property of DSV is attributable to its rich repertoire of metabolites. Therefore, taken together, DSV was found to be capable of inhibiting the entry of the SARS-CoV-2 pseudovirus into human lung epithelial cells. Consequently, DSV could also mitigate the host-pathogen interaction and cytokine upsurge. These properties of DSV substantiate its prophylactic and therapeutic potentials against COVID-19.

## Data Availability

The original contributions presented in the study are included in the article, further inquiries can be directed to the corresponding author.

## Abbreviations

DSV, Divya-Swasari-Vati; ACE 2, Angiotensin Converting Enzyme 2; ATCC, American Type Culture Collection; COVID-19, Coronavirus Disease 2019; DMEM, Dulbecco’s Modified Eagle Medium; ELISA, Enzyme-linked Immunoassay; FBS, Fetal Bovine Serum; HRP, Horse Radish Peroxidase; IL-1β, Interleukin-1β; IL-6, Interleukin-6; MERS, Middle East Respiratory Syndrome; MERS-CoV, Middle East Respiratory Syndrome-related Coronavirus; M^pro^, Main Protease; NCCS, National Centre for Cell Science; RBD, Receptor Binding Domain; RPMI, Roswell Park Memorial Institute Medium; S protein, Spike protein; SARS, Severe Acute Respiratory Syndrome; SARS-CoV, Severe Acute Respiratory Syndrome Coronavirus; SARS-CoV-2, Severe Acute Respiratory Syndrome Coronavirus 2; SEAP, Secreted Embryonic Alkaline Phosphatase; TNF-α, Tumor Necrosis Factor-α; VSV, Vesicular Stomatitis Virus; VSVppSARS-2S, SARS-CoV-2S pseudotyped VSV.
